# Quality of Life and Anxiety Levels in Latin American Immigrants as Caregivers of Older Adults in Spain

**DOI:** 10.3390/healthcare10122342

**Published:** 2022-11-22

**Authors:** Francisco Javier Fernández-Carrasco, Erika Marbely Molina-Yanes, Irene Antúnez-Calvente, Luciano Rodríguez-Díaz, Francisco Javier Riesco-González, Juan Gómez-Salgado, Rocío Palomo-Gómez, Juana María Vázquez-Lara

**Affiliations:** 1 Department of Obstetrics, Punta de Europa Hospital, 11207 Algeciras, Spain; 2 Nursing and Physiotherapy Department, Faculty of Nursing, University of Cádiz, 11207 Algeciras, Spain; 3 Nursing Department, Faculty of Health Sciences of Ceuta, University of Granada, 51001 Ceuta, Spain; 4 Department of Sociology, Social Work and Public Health, Faculty of Labour Sciences, University of Huelva, 21007 Huelva, Spain; 5 Safety and Health Postgraduate Programme, Universidad Espíritu Santo, Guayaquil 092301, Ecuador; 6 Department of Obstetrics, La Linea de la Concepción Hospital; 11300 La Línea de la Concepción, Spain

**Keywords:** anxiety, quality of life, immigrants, caregivers, older people

## Abstract

Increased life expectancy in Spain has highlighted the need for formal caregivers who care for older adults who live at home. In many cases, families choose to hire immigrants from Latin America who, on many occasions, have been forced to emigrate, which causes a considerable psychological impact on them. To this, other factors such as precarious working conditions, loneliness, or high workload are added, which leads to many caregivers becoming sick. The aim of this study was to assess the quality of life and the level of anxiety derived from the workload of Latin American immigrants who come to Spain to care for older adults. A descriptive cross-sectional study was carried out using two validated questionnaires to measure this relationship. A correlation was also established between quality of life and anxiety as expressed by the participants. The sample consisted of a total of 426 subjects. One of the main results showed that the lower the caregiver’s quality of life, the higher their level of anxiety (OR = 1.06; 95% CI). Live-in caregivers had a lower quality of life than people who did not live in the same house as the cared-for person (OR = 2.22; 95% CI). Working long hours and having a high workload was associated with poorer quality of life and higher levels of anxiety. Assessing immigrants who are formal caregivers and providing them with the support that helps to improve their quality of life is suggested to reduce the occurrence of anxiety disorders.

## 1. Introduction

Nowadays, life expectancy in Spain has increased thanks to technological and medical developments, changes in food habits and lifestyle, improvements in material living conditions, education, and last but not least, access to the healthcare system [1]. Older people live longer nowadays and, due to the pathologies that affect their everyday life, they may end up requiring lots of physical care, which translates into the need for a caregiver [1].

Families provide 93% of older adult care, being daughters the main caregivers; however, these numbers are progressively decreasing due to the incorporation of women into the labor market [2]. On the other hand, formal care provided by public administrations only represents 3%. In Spain, the vast majority of older people are looked after by their sons and daughters of working age [3].

As a consequence, formal care is taking up more importance as a job. In this way, it is becoming one of the few possible occupations for immigrant women and one of their main ones. This is how the figure of the live-in caregiver (24 h) appears mostly among Latin American immigrants, defining them as the main caregivers of older adults [4].

Latin American immigrants are one of the preferred groups for this kind of job in Spain, and they have become a pillar for Spanish homes in need of caregivers [5]. In addition, there are certain racial stereotypes that associate Latin women with personality characteristics such as affection and patience, which perfectly meet the standards for care for older people [5].

According to the World Migration Report 2020 of the International Organization for Migration (IOM), the estimated number of international migrants in 2020 was 272 million, representing 3.5% of the world’s population. In 2019, Europe and Asia received about 82 million and 84 million international migrants, respectively, accounting for 61% of the world’s total international migrant population [6]. More and more people of foreign origin are forced to emigrate illegally due to security reasons, extreme poverty, delinquency, or the Latin American political situation, and are compelled to abandon their country of origin and look for a better future in Spain that will allow them to improve economically, protect their own life, and grow as a person [7].

In Spain, on 1 January 2021, the number of immigrants from Latin American origin was 1,612,775 [8]. Spanish population growth has taken place thanks to the foreign population, because if growth depended on the population that currently inhabits the country, population growth in Spain would have been negative [8].

In Spain, only half a million caregivers out of a group of three million people have legalized their professional situation [9]. Eight out of ten of these women are uneducated and have limited resources [9].

One of the factors that predispose families to choose immigrants to care for older people is the fact that the price they charge for their service is significantly lower. Additionally, they provide better results compared to retirement homes, where the price is considerably higher [10].

Immigrants have the need to find a job, which is not an easy task. Another main advantage for Spanish families is that the foreign caregiver usually lacks family ties. This is why they are seen as able to develop their job with a greater dedication to caring for older people [11].

The work of the caregiver often provides them with job stability, and although it is known that it is a low-paid job, it can be attractive for migrants as they can earn more money than others who are engaged in other jobs. Apart from the salary they earn, this job may also give them the possibility to obtain a residence permit, including nationality [12,13].

In this formal immigrant caregiver model, multiple factors are against the foreigner. One of them is the psychological impact, a consequence of migration and of leaving everything they know behind; and their beloved people, for most of them even parents and children, husbands, wives, traditions, food, occupations, and religion [14].

These workers are forced to learn how to cook Spanish food, become used to a routine full of cultural changes, and acquire another way of doing housework. It is hard for these caregivers to adapt to people, customs, and traditions due to the continuous activity of caring for older adults, and being attentive at all times to the needs of their employer, which is especially hard when considering that the vast majority of older people who require this kind of care have Alzheimer’s disease, dementia, or are of advanced age [15]. In Spain, until 2020, 800,000 cases of dementia were known according to the Spanish Society of Neurology [16].

In addition, research shows that the duties and burdens of care that caregivers deal with are associated with both physical and mental health problems. Some associated illnesses include physical problems such as back pain, headache, and muscle pain. Regarding mental health, anxiety, depression, and sleeping disorders may be associated with stress and with the fact that caregivers stop caring for themselves and put their own needs last [16,17,18]. More than half of caregivers feel really tired from caring for their family members, while two in ten are at risk of suffering health-related consequences as a result of the workload. The literature reports that severe fatigue have a prevalence ranging from 49.5% to 76.7% in this population group [17,18,19,20,21]. This is caused not only by the fact of emigrating, the psychological impact, the loneliness, and the level of hard work, but also in many cases by the precariousness of the working conditions and the need to keep their job [22].

The day-to-day life of an immigrant can be hard. Many of them work more than 8 h per day, resting only on Sunday when, usually at night, they return to their job; on many occasions they even have nowhere to go or who to go with. In addition, most foreigners have the need to give economic support to their families back in their home country and this, along with their daily expenses for self-care and rent, becomes barely manageable due to their low salaries. All of this leads to psychological pathologies such as anxiety, somatization, depression, and sometimes suicide attempts [23].

The majority of those who do not work as live-in caregivers may share a room with between two and four people in order to save money by sharing expenses. Thus, this is why working as a live-in caregiver in many cases may appear to be a solution to the living situation though, in the end, the reality is far different from this. Many of these workers suffer from anxiety symptoms due to isolation or their workload [24].

An example of the typical workload that many caregivers carry could be medical visits, accompaniment in hospital stays, hours at the hairdresser, shopping for food, dressing, administration of medication at correct times, collecting medication at the pharmacy, hygiene, walks with the older person to keep them active, on many occasions preparation of meals for visiting family members, diaper changes, measuring glucose for insulin control, and giving injections in case of diabetes disease. If the older adult has limited mobility, their caregivers should make postural changes, manage maintenance and hydration of the skin, cure pressure ulcers if they appear, and report to the employer. Therefore, not only do they perform the role of a caregiver but, in many cases, they are nurses and assistants as well, which conforms them as the right-hand person of the older adult and an important support for home visiting nurses [5].

This whole situation makes the caregiver develop a bond with the older adult and see them as a relative of their own. This way, this type of relationship may become a risk factor for depression. In many cases, immigrants end up suffering unfair dismissals or the death of the older person they are caring for, which leaves the caregiver in the same precarious situation as at the beginning: without a job or opportunities to opt for a different type of job. Within the immigrant population, only a small percentage manages to perform other jobs rather than caring for older adults [25].

Bover et al., studied the quality of life of 514 Latin American women working as caregivers in Spain. Among the main results, they found that the precarious conditions in which these women worked, such as being a live-in worker, the lack of an employment contract, and the multiple household and care tasks they had to carry out, contributed to a low quality of life. The limitations found were that, as they were irregular migrants, there were no records on them so it was difficult to obtain the sample [5]. In another research, Leibach et al., studied the relationship between mental health problems and quality of life among immigrant caregivers with multiple sclerosis, finding that there is a strong association between anxiety and quality of life among these caregivers [26].

So far, it is known that migrant workers who work as caregivers show a high degree of invisibility and social discrimination. It is not known how this affects their health and the degree of vulnerability brought about by the accumulation of certain social and life-conditioning factors. This research contributes to the literature in that Latin American workers are visible as having a poorer quality of life and higher levels of anxiety when working as live-in caregivers and having to perform multiple tasks related to both domestic and care work.

The objective of this research was to assess the quality of life and level of anxiety derived from the workload involved in the job of Latin American immigrants who come to Spain and dedicate themselves to caring for older adults.

## 2. Materials and Methods

### 2.1. Study Design

A descriptive cross-sectional study was designed using questionnaires. The design was based on the following research question: Is there a relationship between the quality of life of Latin American people who come to Spain and dedicate themselves to formal caring and the probability of suffering from anxiety disorders? According to the PECO format, the (P) population is Latin American immigrants who care for older people and people with chronic health problems; the (E) exposure refers to immigrants who have a worse quality of life, mainly those who live with the cared-for person; the (C) control indicates immigrant caregivers who have a better quality of life (not live-in workers); and the (O) corresponds to the outcomes, which are the scores obtained in the Beck Anxiety Inventory. Other variables that were also taken into account were educational level, income, having had a problem in Spain due to being a migrant, and other variables related to workload, such as having to clean the house, wash clothes, cook, feed, help to urinate or defecate, manage hygiene and personal care, help to dress, help to walk or mobilize, and last but not least, prepare and administer medication to the older person. In addition, the following variable was included: ‘Currently taking anxiolytics’.

### 2.2. Participants

The immigrant population from Latin America residing in the cities of Madrid, Barcelona, Seville, and Jerez de la Frontera was studied. They belonged to a group or association in their home country that supported them. Specifically, these associations were Hondusol; Asociación De Hondureños Solidarios, in Barcelona; Asociación Nicaragüita, in Madrid; Asociación Nicas and Asociación Venezolanos Unidos in Andalusia and Spain (Seville); and Asociación Colonca: Colombinos Unidos in Cadiz and Spain, in Jerez de la Frontera, Cadiz.

According to the INE (Spanish National Statistics Institute), the total number of immigrants in Spain in 2021 was 5,375,917 [8]. Of them, only 30% were Latin American. Therefore, the total population of Latin Americans in Spain was 1,612,775. Although the ideal procedure would have been to calculate the sample based on the number of Latin American caregivers working in Spain, when developing the study, it was impossible to obtain these data as there were no associated official records. To calculate the sample size, the Sample Size Calculator program was used. Assuming a reliability of 95% and a margin of error of 5%, a minimum sample size of 385 individuals was needed. Finally, the sample was larger and consisted of 426 subjects. Sampling was done for convenience, non-randomly, and using the snowball technique. It was decided to increase the sample size by 20% in order to assume the possible losses, considering a total of 462. In order for the questionnaire to be considered valid, the subject had to have answered all the questions in the questionnaire. Thus, of the final 426 participants, as 36 people did not answer the questionnaire in full (at least 99% of it), they were discarded, and a total of 426 subjects were finally included. The participants did not receive any financial compensation for their collaboration.

The inclusion criteria established were to be a male or female immigrant over 18 years old, to have arrived in Spain from Latin America, and to dedicate themselves to caring for older adults and people with chronic health problems. People who already suffered from any psychiatric problems such as anxiety, depression, post-traumatic stress disorder, etc., and who had been diagnosed in their country of origin, as well as people whose caregiver role implied less than two hours per day, were excluded.

### 2.3. Instruments

Three questionnaires were used for data collection: one for socio-demographic data and two validated questionnaires: the WHOQOL-BREF quality of life assessment [27] and the Beck Anxiety Inventory (BAI) [28].

For the socio-demographic data questionnaire, a survey was prepared by the authors, in which demographic data were requested such as age, marital status, educational level, income, number of children, country of origin, year of arrival to Spain, how long had they been a caregiver, intake of anxiolytics, and other questions related to the activities they carried out during their working hours.

There are several scales to measure the quality of life, but the WHOQOL-BREF scale was chosen for its good psychometric robustness and fast administration time. Because it is internationally tested in different cultures, it can be self-administered or help can be received for its completion, and it is adapted to its use in Spain. Finally, this scale was chosen because, with it, four dimensions were obtained that could offer valuable information about physical and mental health, (i.e., ‘To what extent do you feel that physical pain prevents you from doing what you need to do?’, ‘To what extent do you feel your life to be meaningful?’), social fulfillment, (i.e., ‘How satisfied are you with your personal relationships?’, ‘How satisfied are you with the support you get from your friends?’), and environmental quality of life, (i.e., ‘How satisfied are you with your access to health services?’, ‘How satisfied are you with the conditions of your living place?’). All of these are factors possibly related to immigrant caregiver overload and, in addition, the scale can also be used to carry out causality studies [27].

The result of the scale is obtained by answering 26 questions with Likert-type answers with scores from 1 to 5. The scoring scale is positive in all questions (a higher score denotes a better quality of life) except in questions 3, 4, and 6, in which a lower score denotes a higher quality of life. The score range is between 26 and 130 and the reliability was tested by measuring Cronbach’s alpha coefficient, which was 0.95. For this sample, the general Cronbach’s alpha coefficient for the complete scale was 0.90.

To measure anxiety, the Beck Anxiety Inventory (BAI) [28] was used. This questionnaire was designed by Beck et al., in 1988, with the aim of reliably discriminating anxiety by assessing its symptoms, (i.e., feeling hot, dizzy, or lightheaded, being nervous, terrified, or afraid, suffering inability to relax, fear of losing control, or difficulty in breathing, among others). It consists of 21 items that are scored using a Likert-type scale from 1 to 5. The score can range from 21 and 105 points and is positively correlated so the higher it is, the greater the level of anxiety of the patient. The author established different severity levels according to the range, considering mild anxiety a score between 21 and 48 points, from 49 to 76 it was classified as moderate, and from 77 to 105 it was classified as severe. It is a questionnaire with high internal consistency and reliability. For this study sample, the reliability of the scale was measured, obtaining a Cronbach’s alpha coefficient of 0.93. This questionnaire was used and adapted to its context in Spain by Sanz [29] and his team in 2012, showing psychometric characteristics similar to the original ones.

### 2.4. Data Collection

Data were obtained between 1 February and 31 March 2022. The researchers contacted the managers of these associations in order to obtain access to their members. The associations provided the researchers with a list of the people who were registered. Then, the researchers telephoned these people and asked them what job they worked for a living. In this way, those who claimed to be working as caregivers were identified. Once the caregivers were identified, they were informed of the purpose of the study and were asked for their consent to participate in it. Those who agreed to participate voluntarily in the study were sent a link by WhatsApp and email so that they could complete the questionnaire from any device with internet access.

### 2.5. Data Analysis

The descriptive analysis of quantitative variables was carried out using measures of central tendency and dispersion. Categorical variables were described in absolute numbers and percentages. In the bivariate statistical analysis, to relate quantitative variables and after applying the Kolmogorov–Smirnov normality test, parametric tests were used. In those variables that followed a normal distribution, Student’s *t*-test was used to compare two independent variables and ANOVA for more than two. Non-parametric tests were used for variables that did not follow a normal distribution. The Mann–Whitney U test was used to compare two independent variables and the Kruskal–Wallis test was used for more than two. Finally, a multivariate binary logistic regression model was developed to establish associations between those independent variables that showed statistical significance in the previous analysis (living with the person they care for, having had problems in Spain due to being a foreigner, having been insulted for being a foreigner, having been physically assaulted for being a foreigner, having felt excluded by Spanish society, helping the cared-for person to move and walk, and anxiety) and the dependent variable (quality of life), which was codified as one for scores below the mean and as zero for scores above it. Confidence intervals (CI) at 95% and a significance level of *p* < 0.05 were obtained. The statistical study was carried out with the IBM SPSS Statistics version 28.

### 2.6. Ethical Aspects

This study adhered to the principles articulated in the Declaration of Helsinki, updated in 2013 in Brazil. This Declaration frames ethical principles to conduct research on human beings. This study is subject to ethical standards that promote and ensure respect for all human beings and protect their health and individual rights. To ensure anonymity, personally identifiable data were replaced by numbers. All the participants signed informed consent. Authorization was obtained from the Research Ethics Committee of Almeria (Spain), with the code TFM-ICDP-2020. The data collected in the study were treated with absolute confidentiality in accordance with the provisions of Spanish laws, specifically Organic Law 3/2018, of 5 December, on the Protection of Personal Data and Guarantee of Digital Rights, and Law 41/2002, of 14 November, on the basic regulation of the autonomy of the patient and rights and obligations in matters of information and clinical documentation.

## 3. Results

### 3.1. Descriptive Analysis

The sample was formed by 426 subjects of which 408 were women (96%) and 18 were men (4%) The rest of the qualitative variables are shown in Table 1. The mean age was 36 years, with a standard deviation (SD) of 8.67, the minimum age being 21 years, and the maximum being 65. Table 2 shows the descriptive analysis of quantitative variables.

### 3.2. Correlational Analysis

Anxiety was negatively correlated with quality of life, so that the lower the person’s quality of life was, the higher their anxiety levels (Table 3).

### 3.3. Comparisons of Means and Bivariate Analysis

The quality of life variable (WHOQOL-BREF test scores) followed a normal distribution, so parametric tests were used. However, the anxiety variable (BAI test scores) did not follow a normal distribution, so non-parametric tests were applied.

#### 3.3.1. Anxiety and Quality of Life According to Income

Statistically significant differences were found when relating the income variable to the anxiety variable (*p* = 0.03). The mean in the BAI test for people who earned less than 700 euros per month was 21.92 (SD = 11.54); for those who earned between 700 and 1500 euros per month, it was 19.86 (SD = 9.78); for those who earned between 1500 and 2000 euros per month, the mean 15.50 (SD = 9.57); and for those who earned more than 2000 euros per month, it was 14 (SD = 10.24). In this way, it was observed that the lower the monthly income, the higher their anxiety levels.

The posthoc test did not establish significant differences between the different groups. Statistically significant differences were not found when comparing the quality of life variable with the income variable.

#### 3.3.2. Anxiety and Quality of Life According to Anxiolytics Intake

In the questionnaire, participants were asked to state what medication they were taking at the moment, and it could be observed that almost 10% of the sample was taking anxiolytics.

Statistically significant differences were found for both associations. People who were taking anxiolytics had poorer quality of life and higher levels of anxiety.

The mean in the WHOQOL-BREF test for people who took anxiolytic medication was 63.17 (SD = 11.54), compared to 71.86 (SD = 9.78) for people who did not take this type of medication (*p* = 0.001).

The mean in the BAI test for people who took anxiolytics was 29.07 (SD = 10.44), while for people who did not take anxiolytics it was 19.45 (SD = 10.22) (*p* = 0.001).

#### 3.3.3. Anxiety and Quality of Life Related to Working as a Live-in Caregiver

It was observed that people who worked as live-in caregivers had more anxiety than those who did not, although no statistically significant differences were found in this association.

Statistically significant differences were found when associating quality of life with the variable working as a live-in caregiver. People who lived in the house of the person they cared for had a lower quality of life (WHOQOL-BREF = 61.65; SD = 10.69) than those who did not (WHOQOL-BREF = 65.24; SD = 11.03) (*p* = 0.001).

#### 3.3.4. Anxiety and Quality of Life Related to Having had a Problem in Spain due to Being a Foreigner

When comparing the anxiety and quality of life variables with having had a problem in Spain due to being a foreigner as a variable, statistically significant differences were found for every association. Thus, people who had had a negative experience in Spain due to being foreigners had a lower quality of life and higher levels of anxiety than people who had not experienced this problem.

The mean WHOQOL-BREF score for people who declared they had not had any problems in Spain due to being a foreigner was 68.03 (SD = 10.96); for people who had been insulted for being a foreigner, it was 59.52 (SD = 8.89); for people who had been at risk of physical harm by another, the mean score was 57.26 (SD = 8.54); and for people who had felt excluded by Spanish society, it was 59.31 (SD = 9.9) (*p* = 0.001). Bonferroni’s posthoc test established that statistically significant differences were found between the group that had had no problem and the group that had been insulted (*p* = 0.001), those who had been physically harmed (*p* = 0.001), and those who had felt excluded by Spanish society (*p* = 0.001).

The mean score in the BAI test for the people who stated not having had any inconvenience due to being a foreigner was 16.44 (SD = 9.48); for people who had been insulted as a consequence of being a foreigner, it was 21.98 (SD = 9.5); for people who had been at risk of physical harm by another, the mean score was 25.35 (SD = 8.79); and for people who had felt excluded by Spanish society, it was 24.3 (SD = 11.68) (*p* = 0.001). The posthoc test established statistically significant associations with those who had not had any problems compared to those who had been insulted (*p* = 0.001), those who had been at risk of physical harm by another (*p* = 0.003), and those who had felt excluded by Spanish society (*p* = 0.001).

#### 3.3.5. Anxiety and Quality of Life Related to Daily Working Hours

People who worked more hours per day had a lower quality of life and higher levels of anxiety.

The mean score in the BAI test for people who worked more than 16 h per day was 22.38 (SD = 10.92); for people who worked from 9 to 16 h per day, it was 20.09 (SD = 9.95); for people who worked from 4 to 8 h per day, the mean score was 17.8 (SD = 10.49); and for people who worked less than 4 h a day, it was 19.05 (SD = 11.33) (*p* = 0.005). The posthoc test confirmed statistically significant differences between the group of people who worked from 4 to 8 h a day and the group of people who worked more than 16 h a day.

The mean WHOQOL-BREF score for people who worked more than 16 h per day was 60.37 (SD = 9.9); for people who worked from 9 to 16 h per day, it was 62.99 (SD = 10.6); for people who worked from 4 to 8 h a day, the mean score was 66.62 (SD = 11.69); and for people who worked less than 4 h a day, it was 66.29 (SD=12.41) (*p* = 0.001).Bonferroni’s posthoc test established that statistically significant differences were found between people who worked from 4 to 8 h (*p* = 0.001), people who worked from 9 to 16 h (*p* = 0.001), and the group that worked for more hours (*p* = 0.001).

As regards to working during holidays and weekends, statistically significant differences were found for the quality of life variable, being the result that people who worked more hours during weekends and holidays had lower levels of quality of life. Bonferroni’s posthoc test established that significant differences between groups were found in the group that worked the most hours (between 9 and 16 h) and the group that worked the least hours during weekends and holidays (less than 1 h) (*p* = 0.01).

#### 3.3.6. Anxiety and Quality of Life Related to Helping in the Daily Life Routines

Statistically significant differences were found between the quality of life variable and anxiety related to having to cook as part of their daily tasks to complete their work activity.

The mean WHOQOL-BREF score for people who had to cook for the person they cared for and for the rest of the family was 60.48 (SD = 10.81), and for people who did not have to cook it was 67.97 (SD = 11.43) (*p* = 0.001).

In relation to the anxiety variable, the mean score for the BAI test for workers who had to cook for the person they cared for and for the rest of the family was 24.39 (SD = 11.78); for workers who cooked only for the person they cared for, it was 19.81 (SD = 10.16); for workers who only prepared breakfast or snacks without including the main meals, the mean score was 19.19 (SD = 8.06); and for workers who did not have to cook, it was 18.03 (SD = 9.26) (*p* = 0.001).

Likewise, workers who had to feed the person they cared for showed a lower quality of life than those who did not, finding statistically significant differences. It was also observed that these caregivers had higher levels of anxiety, although no statistically significant differences were found in this association.

The quality of life and anxiety variables are related to the fact that caregivers have to help the person to urinate and defecate, and statistically significant differences have been found for all the associations. As a consequence, caregivers who had to develop these tasks had a worse quality of life and higher levels of anxiety (WHOQOL-BREF = 61.4; SD = 10.66; *p* = 0.001) (BAI = 18.45; SD = 9.66; *p* = 0.02).

When relating the variables quality of life and anxiety with having to help to move and walk as a variable, statistically significant differences were found for all the associations. In this way, people who had to perform this task had a worse quality of life and higher levels of anxiety (WHOQOL-BREF = 69.7; SD = 12.91; *p* = 0.001) (BAI = 20.99; SD = 10.75; *p* = 0.04).

Regarding having to help the person with cleanliness and personal hygiene as a variable, statistically significant differences were found with the quality of life variable (WHOQOL-BREF = 69.95; SD= 10.66; *p* = 0.001).

Lastly, for having to prepare and administer the medication to the person cared for taken as a variable, statistically significant differences were found for all the associations. In this way, people who had to perform this task had a worse quality of life and higher levels of anxiety (WHOQOL-BREF = 70.09; SD = 12.82; *p* = 0.002) (BAI = 20.97; SD = 10.89; *p* = 0.03).

### 3.4. Multivariate Analysis

In order to carry out the multivariate analysis, a binary logistic regression was performed. Those variables that showed significance in the bivariate analysis were included as independent variables. The model properly fits Hosmer and Lemeshow Chi-squared = 23.72 (*p* = 0.01) and Nagelkerke’s R-squared = 0.49 (Table 4).

## 4. Discussion

Being a caregiver in Spain is full of sacrifices, resignation, and very hard moments, not only in the home and private spheres, but also in the professional one [9]. In Spain, there are three million caregivers [30]. In our study, the vast majority of the sample (98%) was made up of women. They were mainly women who did not have a work permit in Spain and who provided care for people in an irregular situation. Men were only represented by 2%. This may be attributed to the fact that the male population tends to find job opportunities in other types of jobs, mostly in the service sector, and they do not dedicate themselves to caring for people with chronic health problems or older adults [31].

Caring for older adults with dementia is associated with a well-documented increased burden of care and distress, and decreased mental health and well-being. The most severe behavioral, cognitive, and functional impairments in a patient are associated with higher levels of burden and distress. Distress increases as hours of care per week and the number of tasks performed increases, and as coping resources and support decrease [32].

Among the main findings of the present study, it has been observed that quality of life is negatively correlated with anxiety. It has also been found that those who took anxiolytics had a lower quality of life and higher levels of anxiety. Many studies report that formal care is a source of chronic stress that can have serious consequences on physical and mental health [33,34,35].

Another significant finding is that 68% of the studied population showed moderate or severe levels of anxiety according to the Beck Anxiety Inventory. Soltys and Tyburki [35] reported in their research that caregivers usually have high levels of anxiety. These immigrants, on many occasions, live in the home of the people they care for [31]. In the present study, this group was made up of 63%.

Data revealed in this study showed that people cohabiting with the person they cared for had lower quality of life. The multivariate analysis also revealed that people who were live-in caregivers had more than double the risk of having a worse quality of life. Roberson et al. [36], in a systematic review, reported similar results.

In Spain, episodes of xenophobia have been reported. The discrimination perpetrated against people because they belonged to another ethnic group, whether they are immigrants or autochthonous, has well-known consequences on health [36]. More than half of our sample declared problems in Spain due to being foreigners: 27% had been insulted, 25% had felt excluded by Spanish society, and 5% had been attacked or had been at risk of an attack for the mere fact of being a foreigner. The multivariate study revealed that people who had problems due to being a foreigner had a 6.36 times greater risk of having a worse quality of life [37].

Health outcomes as an effect of experiences of racism have been studied fundamentally through mental health, especially as regards to anxiety [38,39]. The crisis due to the COVID-19 pandemic has also affected immigrants, who felt more excluded by Spanish society when wanting to protect themselves against the virus [40,41].

An association was identified between the quality of life and the number of working hours immigrants spent, as well as with the work overload derived from activities such as preparing food, having to feed the person they care for, helping the person to urinate and defecate, helping the person to move and walk, cleaning the person, and preparing and administering medication. Several studies coincide with these results [35,42].

Anxiety was negatively correlated with quality of life. In this study, anxiety was significantly associated with having had some inconvenience due to being a foreigner, with the excessive number of working hours, and with the overload of tasks such as having to cook, having to help the person they care for urinate and defecate, having to help the person to move or dress, or having to prepare and administer the medication to the person cared for. Briones-Vozmediano et al. [43] carried out a multicenter qualitative analysis in Spain to describe the consequences on the health of immigrant women who care for older adults. Among the negative consequences, anxiety and depression were described.

Another relevant aspect affecting anxiety levels was income. People who earned a lower salary had more anxiety than those who received higher salaries. Hewko et al. [44] define this work context as marginalizing, showing that live-in caregivers are the worst paid; they officially work fewer hours and obtain less benefit from their work, even when compared to other health professionals.

There are several limitations in the present study which deserve to be mentioned. Though it would have been interesting to include certain variables, no relevant data were obtained on the chronic illnesses of the cared-for people or the length of time the caregivers had been working as such. Another limitation could be associated with the way the sample was obtained. Various immigrant associations were contacted in order to pass the questionnaire to their associates. As a consequence of that, the sample was not chosen randomly, but was made up of all the people who wanted to participate in the study voluntarily and who met the inclusion criteria. That is why, despite being representative in number, caution must be taken when generalizing the results. Another aspect to mention is that data were self-reported by the participants, which could lead to biases such as forgetfulness, selective memory, or exaggeration. It should also be acknowledged that assessing anxiolytic medication was a way to measure the use of pharmacological treatment for anxiety, so findings should be interpreted cautiously. Finally, multiple linear regression was not applied because the criteria for normality of quantitative variables in the model were not met and there was high collinearity, so it was decided to perform a binary logistic regression analysis instead. This should be kept in mind as, normally, continuous quality of life scores should have been analyzed with a multivariate regression model instead of dichotomizing them. The categorization of continuous variables leads to loss of information, may significantly underestimate the variability, and may ultimately lead to biased conclusions [45], so the authors recommend estimating these results with caution.

Considering migration, a powerful social determinant of health means that the findings of the study point to the need to monitor the health conditions of these vulnerable groups, such as Latin American immigrant women working as caregivers, though the outcomes have certain limitations inherent to research with an immigrant population due to the lack of records that make it difficult to obtain the sample. This has become even more dramatic given the socio-economic and political changes that have restricted access to social and healthcare resources and the labor market, and that has occurred in Spain since the study data were collected, increasingly affecting a progressively larger population. In light of this, policies to support the health of the migrant population should be strengthened with multisectoral strategies, from a gender perspective and linked to the family structure. These strategies should reinforce specific programs to empower women who have recently arrived in the country or are in an irregular situation, and especially those who need to improve their working conditions, thus making it possible to reduce inequalities in health.

As health care for older adults increasingly takes place in the home of the cared-for person, caregivers represent the key emblem of care provision, and they require greater preparation to provide such care [46]. Implicit assumptions behind this policy are that reductions in formal long-term care in hospital settings will be compensated by increased informal (and formal) domestic care [47]. This is a key factor that requires effective preparation, strong inter-institutional coordination, high-quality communication, and the assumption of shared goals, knowledge, and mutual respect [46].

## 5. Conclusions

It has been observed that being a live-in caregiver of older people affects the immigrant worker. This could be explained by the many hours the work implies, the few hours of restful sleep, and that there is only one rest day per week, resulting in an alteration of the quality of life and thus producing mental disorders.

The results of this study invite reflection for the support that dependent people receive from health services. They also shed light on the masked reality of migrant caregivers that can go unnoticed due to the vulnerability inherent to this group.

The great overload the caregiver has should also be considered as, in addition to the fact that they have to care for the person 24 h per day, they are also in charge of other activities such as cooking, ironing, washing, shopping, medicating, accompanying to consultations, helping with hygiene and personal care, providing emotional support, etc. All of this leads to the previously outlined overload that affects the quality of life of the immigrant caregiver.

Looking to the future, it is necessary to make an adequate assessment of the quality of life of immigrant workers in order to provide support and improve their quality of life so that they may have lower levels of anxiety. More attention should be paid to the immigrant formal caregiver group to make a prompt and adequate evaluation of the relationship between quality of life and anxiety disorders, given the fact that they conform to a highly vulnerable group due to the living and working conditions they have in Spain.

As a result of this study, further research on this problem is proposed in order to improve not only the quality of life of immigrant caregivers but of older adults as well.

## Figures and Tables

**Table 1:** Descriptive analysis of qualitative variables.

		*n* (*N* = 426)	%
Sex	Man	18	4
	Woman	408	96
Marital status	Single	257	60.3
	Married	98	23
	Divorced	68	26
	Widower	3	0.7
Incomes	Less than 700 euros per month	162	38
	Between 700–1500 euros per month	239	56.1
	Between 1500 and 2000 euros per month	20	4.7
	More than 2000 euros per month	5	1.2
Children	Have no children	179	42
	Have children born in their home country	205	48.1
	Have children born in Spain	24	5.6
	Some of the children were born in their country of origin and some were born in Spain	18	4.2
Have commitments to support the family in their home country	Yes	316	74.2
	No	110	25.8
Work internally in the home of the person cared for	Yes	270	36.6
	No	156	63.4
Have had problems in Spain due to being a foreigner	Have had no problems	182	42.7
	Have been insulted	115	27
	Have been at risk of suffering harm from others	23	5,4
	Have felt excluded by Spanish society	106	24,9
Currently taking anxiolytics	Yes	42	9.9
	No	384	90.1
Daily working hours	Less than 4 h per day	21	4.9
	Between 4–8 h per day	95	22.3
	Between 9–16 h per day	149	35
	More than 16 h per day	161	37.8
Working hours on weekends and holidays	Less than 1 h	84	19.7
	Between 1–3 h	72	16.9
	Between 4–8 h	104	24.4
	More than 9 h	166	39
House cleaning	Yes	413	96.9
	No	13	3.1
Laundry	Yes	390	91,5
	No	36	8.5
Cooking	No	37	8.7
	Just breakfast and/or snacks	26	6.1
	Just for the person cared for	259	60.8
	For the whole family	104	24.4
Help feeding	Yes	231	54.2
	No	195	45.8
Help to urinate or defecate	Yes	288	67.6
	No	132	32.4
Help to walk or move	Yes	340	79.8
	No	86	20.2
Personal hygiene help	Yes	365	85.4
	No	62	14.6
Help to dress	Yes	373	87.6
	No	53	12.4
Prepare and administer medication	Yes	353	82.9
	No	73	17.1

**Table 2:** Descriptive analysis of quantitative variables.

	*N*	Minimum	Maximum	Mean	Standard Deviation
Age	426	21	65	36.19	8.68
WHOQOL-BREF test score	426	34	113	71	13.28
Beck Anxiety Inventory test (BAI) score	426	0	57	20.39	10.62

**Table 3:** WHOQOL-BREF and BAI test correlation.

		Total WHOQOL-Bref Test
Total BAI test	Correlation coefficient *	−0.55
	Sig (bilateral)	0.001
	*N*	426

* Spearman’s rho correlation coefficient.

**Table 4:** Binary logistic regression analysis.

Independent Variables	OR	95% C.I.	
		Lower	Higher
Living internally in the home of the person cared for	2.22	1.32	3.75
Not having any problems in Spain due to being a foreigner	Ref.		
Having been insulted for being a foreigner	6.36	1.99	20.32
Having been physically assaulted for being a foreigner	3.49	1.94	6.29
Having felt excluded by Spanish society	3.02	1.62	5.66
Helping the person cared for to move and walk	2.77	1.44	5.33
Anxiety	1.062	1.02	1.1

*Dependent variable*: quality of life (0 = adequate quality of life, 1 = bad quality of life). *Independent variables*: age, income, taking anxiolytics (yes/no), the fact of living internally with the person cared for (yes/no), having had problems in Spain due to being a foreigner (no problem/having been insulted/having been physically assaulted/having felt excluded by Spanish society), daily working hours, having to work hours on weekends and holidays, having to cook in the house they work at, having to help to feed the person cared for (yes/no), having to help the person cared for to urinate and defecate (yes/no), having to help the person cared for to move and walk (yes/no), having to help the person cared for with their personal hygiene (yes/no), and anxiety.

## Data Availability

All data are available within this article.
